# Supplementary material for: Re-Engineering Therapeutic Anti-Aβ Monoclonal Antibody to Target Amyloid Light Chain
Source: Int J Mol Sci. 2024 Jan 27;25(3):1593. doi: 10.3390/ijms25031593 (PMC10855199; doi:10.3390/ijms25031593)
Supplement: Supplementary file 1 [file ijms-25-01593-s001.zip › ijms-2800759-supplementary.pdf]

Supporting information

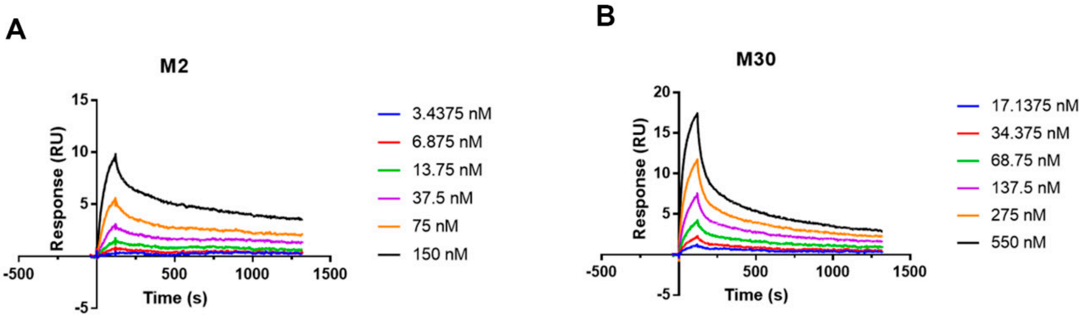

Figure S1. Further affinity analysis of M2 and M30 mutants binding to Aβ1-42 oligomers by Biacore T200. (**A**) M2; (**B**) M30.

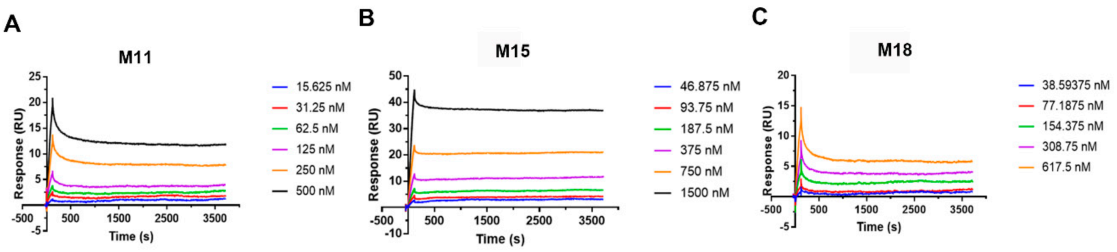

Figure S2. Further affinity analysis of M11, M15 and M18 mutants binding to AL fibrils by Biacore T200. (**A**) M11; (**B**) M15; (**C**) M18.

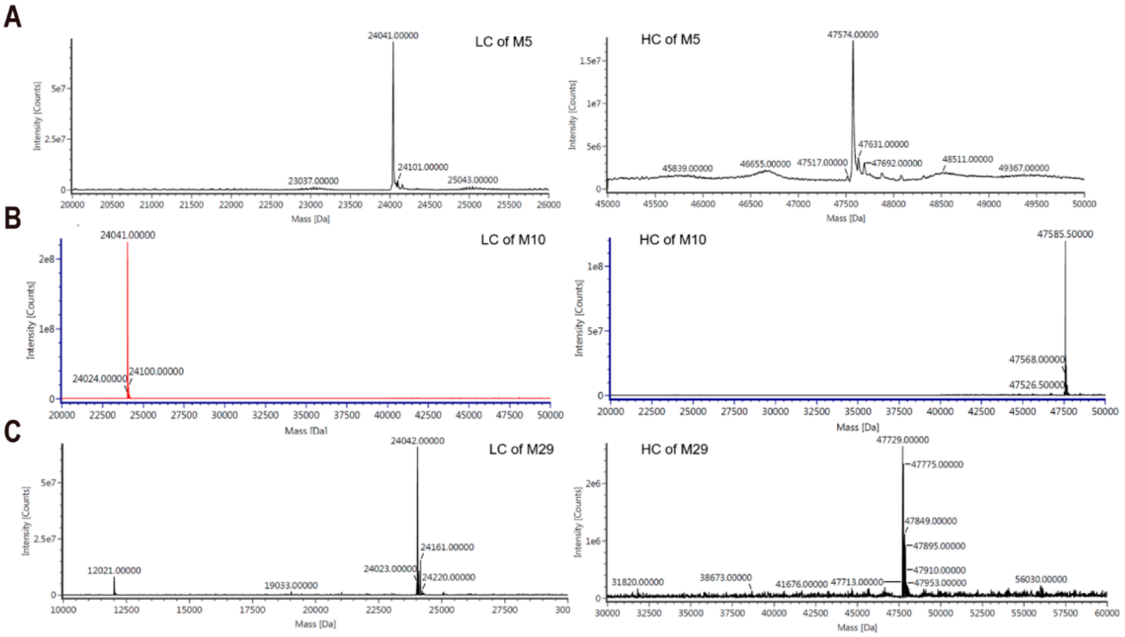

Figure S3. Light chain and heavy chain molecular weight analysis of M5, M10 and M29 by Waters ACQUITY UPLC H-Class system. (**A**) M5; (**B**) M10; (**C**) M29.

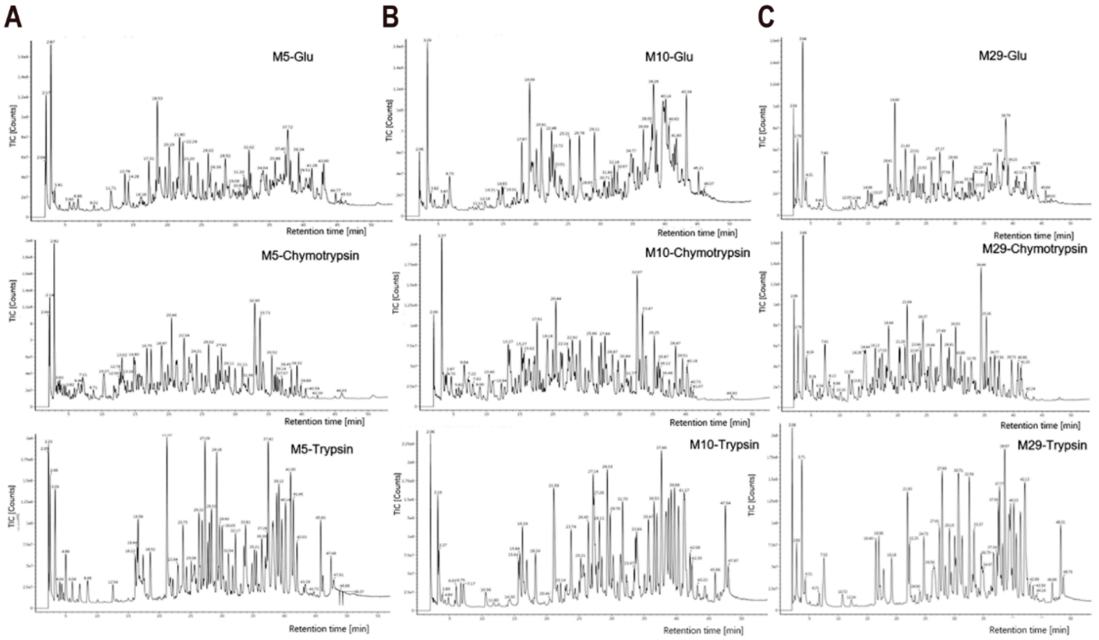

Figure S4. Peptide mapping of M5, M10 and M29 digested by three enzymes (Trypsin, Chymotrypsin, Glu-C) respectively by Waters ACQUITY UPLC H-Class system.
